# Optimizing the Sensitivity of a Pelvic Sentinel Node Algorithm Requires a Hybrid Algorithm Combining Indocyanine Green Based Mapping and the Removal of Non-Mapped Nodes at Defined Anatomic Positions

**DOI:** 10.3390/cancers16183242

**Published:** 2024-09-23

**Authors:** Michele Bollino, Barbara Geppert, Petur Reynisson, Celine Lönnerfors, Jan Persson

**Affiliations:** 1 Division of Gynaecologic Oncologic Surgery, Department of Obstetrics and Gynaecology, Skåne University Hospital Lund, 22185 Lund, Sweden; 2 Department of Clinical Sciences, Obstetrics and Gynaecology, Faculty of Medicine, Lund University, 22185 Lund, Sweden

**Keywords:** pelvic sentinel node, endometrial cancer, hybrid algorithm

## Abstract

**Simple Summary:**

Present algorithms for the detection of pelvic sentinel lymph utilizing only tracers may fail to detect metastatic disease as women with nodal metastases overall have an overall lower mapping rate and that nodes at certain anatomic positions of the pelvic lymphatic pathways map less frequently. The study aimed to assess the increase in the diagnosis of pelvic nodal metastases by adding the removal of non-mapped nodes at defined known high-risk anatomic positions despite mapping at other positions. A 4.3% increase in the detection of pelvic nodal metastases was noted by the presence of isolated non-mapped metastatic nodes in the proximal obturator or external interiliac positions, suggesting that a hybrid algorithm combining tracer (ICG) with the removal of non-mapped nodes at either of the aforementioned positions should be utilized.

**Abstract:**

Aim of the study: to investigate the incidence of non-mapped isolated metastatic pelvic lymph nodes at pre-defined anatomical positions. Patients and Methods: Between June 2019 and January 2024, women with uterine-confined endometrial cancer (EC) deemed suitable for robotic surgery and the detection of pelvic sentinel nodes (SLNs) were included. An anatomically based, published algorithm utilizing indocyanine green (ICG) as a tracer was adhered to. In women where no ICG mapping occurred in either the proximal obturator and/or the interiliac positions, defined as “typical positions”, those nodes were removed and designated as “SLN anatomy”. Ultrastaging and immunohistochemistry were applied to all SLNs. The proportion of isolated metastatic “SLN anatomy” was evaluated. Results: A non-mapping of either the obturator or interiliac area occurred in 180 of the 620 women (29%). In total, 114 women (18.4%) were node-positive and five of these women (4.3%) had isolated metastases in an “SLN anatomy”, suggesting a similar lower sensitivity of the ICG-only algorithm. Conclusion: In an optimized SLN algorithm for endometrial cancer, to avoid undetected nodal metastases in 4.3% of node-positive women, if mapping fails in either the proximal obturator or interiliac area, nodes should be removed from those defined anatomic positions, despite mapping at other positions.

## 1. Introduction

Sentinel lymph node (SLN) mapping has been progressively incorporated into the management of women with endometrial cancer (EC) during later years. The insight that a cervical injection of tracer could accurately identify SLNs has contributed significantly to the advancement of the SLN concept, and indocyanine green has emerged as the tracer of choice [1,2,3,4,5]. The use of ultrastaging and immunohistochemistry has led to improved detection of small-volume metastases [6,7,8,9]. Four prospective studies on SLN detection in primarily high-risk EC where a completory pelvic and para-aortic lymph node dissection was performed demonstrated the potential of an ICG-based SLN concept in EC with a pooled sensitivity to detect pelvic nodal metastases of 95.7% (95% CI, 90.2–98.6) [6,10,11,12]. In the SHREC study, despite high sensitivity, node-positive women had a lower mapping rate compared to node-negative women (8/54 compared with 6/203, *p* < 0.001), particularly in the obturator fossa (22/54, 40.7%) [6]. A similar correlation between nodal metastases and non-mapping was later published by Raffone et al. [13]. In a recent study by Bollino et al., the obturator fossa was found to harbor 49.1% of all SLN metastases and was the sole position of SLN metastases in 25.3% of node-positive women with EC, underlining the importance of recognizing the frequent presence of parallel lymphatics along the upper paracervical pathway [14]. These findings are an important indicator that mapping by a tracer may not be sufficient for detecting all women with pelvic nodal metastases. In addition, not surprisingly, the overall number of metastatic pelvic nodes was found to be higher in women with non-endometrioid histology and/or myometrial depth invasion compared with low-grade tumors with no depth invasion. The number was five (range 1–36) compared with two (range 1–9), indicating that SLN sensitivity data from studies on women with a high-risk EC may not be directly transferable to women with low-risk EC, which indicates a need for a sharpened algorithm [6]. An optimal SLN algorithm in EC must take this into consideration [15]. Nodal status guides adjuvant treatment and affects patient prognosis [16,17,18,19,20,21,22]. It remains uncertain whether a complete pelvic and para-aortic lymphadenectomy (LND) is solely diagnostic or provides a survival advantage [23,24,25,26,27,28,29]. Given previous results regarding the anatomical location of metastatic SLNs, a selective removal of nodes located at these positions is an alternative to a full side-specific lymph node dissection in case of non-mapping [14,30]. More extensive lymph node dissection and associated lymphatic complications are particularly important to avoid in women with low-risk EC, women in whom nodal assessment was previously not performed [6,11,31,32,33,34,35].

The aim of this prospective study was to study the incidence of pelvic nodal metastases in non-mapped typically positioned nodes in the proximal obturator fossa and the interiliac area (“SLN anatomy”).

## 2. Materials and Methods

Between June 2019 and January 2024, women with uterine-confined EC of all risk groups including endometrial intraepithelial neoplasia (EIN) deemed suitable for robot-assisted surgery and fit for adjuvant treatment were offered participation in this modified “SLN-only” study (clinicalTrials.gov NCT03838055). All women with EC underwent a preoperative CT scan of the thorax and abdomen. Women with EIN in whom EC was diagnosed at final histology had a postoperative CT scan. Vaginal ultrasonography was used for preoperative estimation of myometrial and cervical invasion. Inclusion/exclusion criteria are outlined in the online study protocol (Supplementary Text S1). Enrolled women were scheduled for a robotic hysterectomy, bilateral salpingoophorectomy, the detection of pelvic SLNs, and, if indicated, an infracolic omentectomy. A da Vinci^®^ Si or Xi Surgical robot with the FireFly^®^ application was used (Intuitive Surgical, Sunnyvale, CA, USA). The procedures were performed by either of three initial or two gradually introduced surgeons, all of whom were supervised by the surgeon responsible for study protocol (JP). One of the three initial surgeons acted as an assistant or supervisor during all procedures. All SLN procedures abided by a previously published anatomically based surgical algorithm with a strict definition of SLNs [15,36]. A total of 1 mL 2.5 mg/mL ICG solution was injected submucosally in the cervix at 2-4-8 and 10 o’clock (0.25 mL per position) with an ipsilateral submucosal reinjection of an additional 0.25 mL ICG solution at 3 and/or 9 o’clock in case of non-mapping. SLNs along the upper paracervical pathway (UPP) were identified, including SLNs along parallel lymphatics to the obturator and external iliac areas when present and the removal of the parauterine lymphovascular tissue (PULT). The PULT (in the original publication called “The upper paracervical lymphovascular tissue”) is defined as the tissue between the broad ligament and the obliterated umbilical artery, caudal to the supravesical artery, and ventral to the ureter. The PULT contains lymphatic tissue connecting the uterus with the lateral pelvic lymph nodes. [37,38]. Typical positions were defined as the “proximal obturator fossa” (the proximal third of the obturator fossa, lateral of the obliterated umbilical artery, dorsomedial of the ventral rim of the external iliac vein, and ventral of the obturator nerve) and the “interiliac position” (lateral of the obliterated umbilical artery, ventromedial of the external iliac vein, and within the bifurcation of the external and internal iliac arteries). These positions are reached by exploring the “lateral paravesical and pararectal spaces” [38]. In case of non-mapping in either of, or both, the proximal obturator or interiliac positions, nodes located at these positions were removed, designated as “SLN anatomy” and processed as SLNs. All SLN tissue was embedded and bisected if the minimum thickness exceeded 3 mm. Ultrastaging using hematoxylin and eosin staining (H&E) was performed in five sections at two to three different levels, 200 µm apart if the maximum diameter of the sentinel node tissue exceeded 1 mm. Immunohistochemistry with staining for pan-cytokeratin (cytokeratin MNF 116) was performed at one or two levels. Non-SLNs with a thickness of less than 3 mm were embedded entirely, and for nodes exceeding 3 mm, at least half the node was embedded. Non-SLNs were stained for H&E but were not subjected to immunohistochemistry. Metastatic disease was classified according to a modification of the American Joint Committee on Cancer staging definitions for axillary nodes in breast cancer (macrometastases = tumor greater than 2.0 mm in diameter, micrometastases = tumor cell aggregates between 0.2 and 2.0 mm in diameter, isolated tumor cells = individual tumor cells or aggregates that are less than 0.2 mm in diameter and less than 200 cells) [39]. Clinic demographic data and positions and types of SLNs and metastatic SLNs were continuously entered into a database. Descriptive data are presented with numbers and percentages. The study was approved by the Institutional Review Board (Skåne University Hospital, Dnr 2018/541) and registered at Clinical Trials.gov (NCT03838055). Written informed consent was obtained from all enrolled women.

## 3. Results

**Figure 1:**
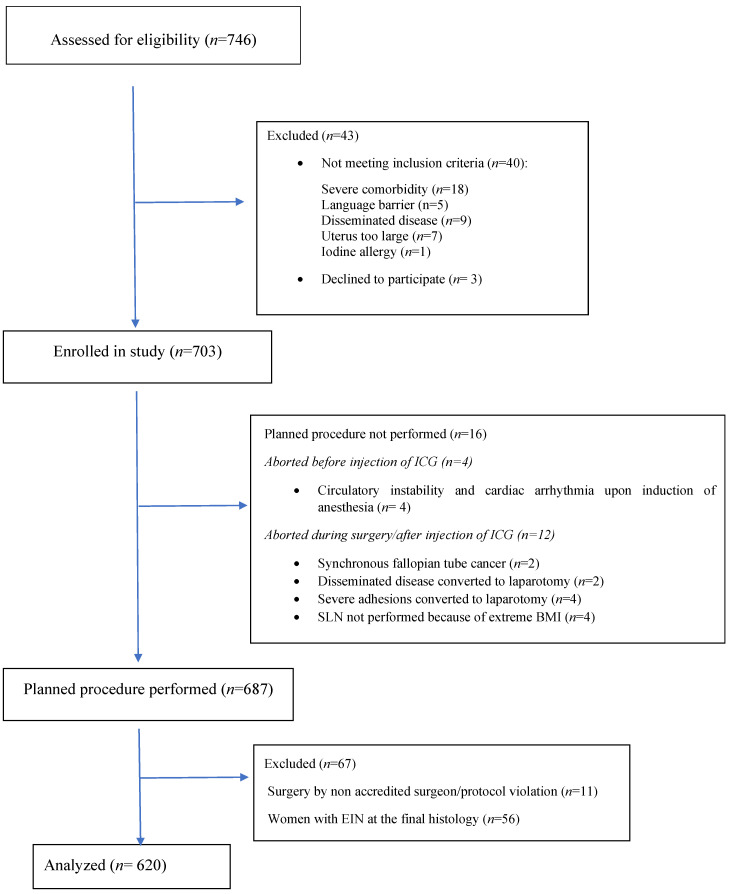
Strobe flow chart of consecutive women with endometrial cancer evaluated for eligibility in a prospective study assessing the proportion of non-mapped metastatic SLN nodes at defined anatomic positions (SLN anatomy).

A total of 746 women were assessed for eligibility, 703 women were enrolled, and data from 620 women were included in the final analysis (strobe flow chart. Figure 1). Demographic and clinical data are presented in Table 1. In women with EC at final histology, 451/620 (72.7%) had a low grade, 50/620 (8.1%) had high-grade endometrioid cancers, and 119/620 (19.2%) had non-endometrioid cancers.

The bilateral ICG-defined mapping rate (at least one ICG-defined SLN per hemipelvis) following the reinjection of tracer in 105/620 (16.9%) women was 93.2% and 87.7% in SLN-negative and SLN-positive cohorts, respectively. The median number of ICG-defined SLNs was 4 (range 1–8) as perceived by the surgeon and 6 (1–20) on final histology. A total of 114 (18.4%) women had pelvic SLN metastases; 14 of these women had metastases in an “SLN anatomy”; in 5 women (4.3%), this was the only manifestation of nodal spread. Of all women with metastatic SLNs, 55/114 (48.2%) had at least one “SLN anatomy” identified and removed due to non-mapping. In 41 (35.9%) women, SLN anatomy was identified in the obturator fossa, and in 30 (26.3%) women, in the interiliac area. Fourteen women had “SLN anatomy” at both locations. In the obturator fossa, 14/41 (34.1%) of “SLN anatomy” were metastatic, and isolated metastases were found in 3/114 (2.6%) of node-positive women. At the external interiliac area, 10/30 (33.3%) “SLN anatomy” were metastatic, and 2/114 (1.7%) of node-positive women had isolated interiliac metastases (Table 2). Of all metastatic SLN anatomy, 10/17 (58.8%) were isolated tumor cells (ITC), 1/17 (5.9%) were micrometastases (MIM) and 6/17 (35.3%) were macrometastases (MAM).

Three of the five women with isolated metastases in an “SLN anatomy” had grade 1 endometroid cancers, and two had non-endometroid cancers. Per intraoperatively utilized protocols, no injuries to the obturator nerve, ureters, or vascular injuries requiring suturing or repair occurred during the removal of SLNs as such. In summary, “SLN anatomy” increased the detection of pelvic nodal metastatic disease by 4.3%.

## 4. Discussion

Removing nodes at typical positions, i.e., “SLN anatomy” identified five node-positive women in addition to the 109 identified by the ICG algorithm, i.e., demonstrating a lack of sensitivity in the range of 5% using ICG mapping only. Three of these had presumed low-risk cancer and were identified using ultrastaging or immunohistochemistry which would go undetected if abiding by traditional algorithms. The remaining two were women with non-endometroid cancers with macrometastases in which non-mapping was probably due to blocked lymphatics because of tumor aggregates. The results from the present study strongly suggest that to optimize sensitivity, an SLN algorithm should, even in the presence of bilateral mapping, be a hybrid between ICG-based mapping and the removal of non-mapped nodes at defined high-risk anatomic positions. This underlines the suggestion in a previous study recommending the removal of nodes at those typical positions, rather than a side-specific lymphadenectomy, in case of a complete hemipelvic non-mapping. This approach helps retain information on nodal involvement while reducing the rate of associated surgical and lymphatic complications [14]. Contrary to most other SLN algorithms, the present study underlines the importance of the exploration of parallel lymphatics within the upper paracervical pathway, i.e., both the interiliac and obturator area [15,30]. Recent studies have shown that the obturator fossa harbors a majority of nodal metastases and a high rate of isolated metastases, which further stresses the significance of exploring this less accessible area to optimize the nodal detection rate even in case of non-mapping [14]. Identifying all women with nodal involvement is important in order to determine the individual woman’s prognosis and to guide the need for adjuvant treatment to optimize care, although a correct assessment also allows for the performance of future studies aiming to further individualize cancer care. Despite the current FIGO staging not taking into account isolated tumor cells in lymph nodes and a clinical situation where there is limited data on the impact on recurrence and survival, we believe it is important to also present data from those women hence considered node-positive in this study. The strengths of this study are the prospective design and the low proportion of protocol violations. Adherence to a strict surgical protocol when performing the procedures and supervision by one initially, and later three accredited surgeons, resulted in a high internal validity. Another strength is the uniform histological management of SLNs consistently applying ultrastaging and immunohistochemistry on all SLNs. Weaknesses of the study are a lack of generalizability of the results and that the results may not be transferrable to other surgical approaches or tracers. Not including the exploration of the lower paracervical pathway (LPP) in all patients contributed to a lower bilateral mapping rate compared to the SHREC study, although the rate of 93.2% in the present study exceeds that of the SENTOR, applying a reinjection of tracer, and the FIRES study where the bilateral mapping rate was 77% and 52%, respectively [10,11]. “SLN anatomy” is in our opinion a complement to and not a replacement of an ICG-based algorithm. Anatomical variations including tortuous vessels seen in many elderly women and SLNs obscured by fatty tissue in women with obesity are challenges where ICG facilitates SLN detection [40,41]. When tracers with proven lower mapping rates such as radiotracers and patent blue are utilized, a hybrid algorithm including “SLN anatomy” might be particularly helpful to optimize the detection of nodal disease without the need for a more extensive nodal dissection [1].

## 5. Conclusions

Non-ICG mapped lymph nodes in the proximal obturator and/or the interiliac anatomic positions contain isolated metastases in 4.3% of node-positive women with endometrial cancer despite mapping at other pelvic positions uni- or bilaterally. Optimizing the sensitivity of the pelvic SLN algorithm should therefore include the removal of non-mapped nodes at those typical positions even when other ICG-mapped SLNs are present at a hemipelvis, i.e., utilizing a hybrid SLN algorithm as described.

## Figures and Tables

**Table 1:** Clinicopathologic characteristics of 620 consecutive women with endometrial cancer included in a study evaluation of an SLN algorithm with the removal of non-ICG mapped lymph nodes at defined anatomic positions in addition to ICG-defined SLNs.

Variable n (%) or Median (Range) as Appropriate	Node Negative Cohort (n = 504)	Node Positive Cohort (n = 116) ^a^	*p* Value ^b^
Age (years)	69 (31–95)	77 (41–88)	<0.01
Body mass index (kg/m²)	28.7 (17.6–66.1)	27 (16.6–47.5)	<0.01
Final histology			
Low grade endometrioid adenocarcinoma (grade I-II)	384 (76.2)	67 (57.7)	<0.01
High grade endometrioid adenocarcinoma (grade III)	37 (7.3)	13 (11.2)	0.23
Serous adenocarcinoma	45 (8.9)	22 (18.9)	<0.01
Clear cell adenocarcinoma	14 (2.8)	6 (5.2)	0.30
Carcinosarcoma	21 (4.2)	7 (6)	0.53
other	3 (0.6)	1 (0.8)	1.0
Stage (FIGO 2009)			
IA	382 (75.8)	-	
IB	90 (17.8)	-	
II	23 (4.6)	-	
III A-B	5 (0.9)	-	
IIIC1	-	89 (76.7)	
IIIC2	-	27 (23.3)	
IVA-B	5 (0.9)	0	
Uterine stage (Irrespective of overall surgical stage)			
IA	397 (78.7)	48 (41.7)	<0.01
IB	107 (21.3)	67 (58.3)	<0.01
II	31 (6.1)	21 (17.9)	<0.01
Lymph node assessment			
SLN biopsy only ^c^	455 (90.3)	68 (58.6)	<0.01
SLN biopsy only ^c^ + PALND	49 (9.7)	48 (41.4)	<0.01

FIGO, International Federation of Obstetrics and Gynecology; PALND, para-aortic lymph node dissection. ^a^ Including 2 women with isolated para-aortic metastases ^b^ Comparisons between groups were evaluated using the two-sample *t*-test for age and body mass index and the χ^2^ or Fisher’s exact test for each of the categorical variables ^c^ Includes detection of SLNs along the LPP (Lower Paracervical Pathway) in women with non-endometrioid histology.

**Table 2:** Overview of positions (all and isolated) of metastatic SLNs defined by indocyanine green (SLN-ICG) or non-mapped lymph nodes at predefined typical positions (“SLN anatomy”) in 114 node-positive women with endometrial cancer.

	Upper Paracervical Pathway		
	External Iliac Area	Obturator Area	Parauterine Lymphovascular Tissue
SLN ICG	45/114 (39.5%)	84/114 (73.7%)	15/114 (13.2%)
SLN ICG isolated	14/114 (12.3%)	25/114 (21.9%)	2/114 (1.7%)
“SLN anatomy”			
	10/114 (8.8%)	14/114 (12.3%)	-
	10/30 (33.3%) *	14/41 (34.1%) *	
“SLN anatomy” isolated			
	2/114 (1.7%)	3/114 (2.6%)	-

Compartments presented independently from each other, implying that each of the 114 node-positive women can have metastatic SLNs, per definitions, in multiple compartments. * Proportion of metastatic SLN anatomy per position.

## Data Availability

All data are available upon request.

## Supplementary Materials

The following supporting information can be downloaded at https://www.mdpi.com/article/10.3390/cancers16183242/s1, Text S1: Online study protocol. References [42,43] are cite in Supplementary Materials.
