# Supplementary material for: Optimizing the Sensitivity of a Pelvic Sentinel Node Algorithm Requires a Hybrid Algorithm Combining Indocyanine Green Based Mapping and the Removal of Non-Mapped Nodes at Defined Anatomic Positions
Source: Cancers (Basel). 2024 Sep 23;16(18):3242. doi: 10.3390/cancers16183242 (PMC11430411; doi:10.3390/cancers16183242)
Supplement: Supplementary file 1 [file cancers-16-03242-s001.zip › cancers-3170723-supplementary.pdf]

Title page

Sentinel node detection in low and high risk endometrial cancer. A prospective observational study.

Table S1. Site information.

Site		Planned enrollment
Department of OB&G<br>Skåne University Hospital, Lund<br>SE 221 85, Lund Sweden		1000-1500 women
**Principal investigator**<br>Jan Persson MD, PhD, Professor<br>Department of OB&G<br>Skåne University Hospital, Lund<br>SE 221 85, Lund Sweden<br>(Ph) +46733522080<br>Jan.persson@med.lu.se		
**Study Coordinator**<br>Michele Bollino MD<br>Department of OB&G<br>Skåne University Hospital, Lund<br>SE 221 85, Lund Sweden<br>(ph)+46707490492<br>Michele Bollino@med.lu.se		
**Study Statistician**<br>Filip Mörk<br>Faculty of engineering<br>Lund University<br>FilipMork@outlook.com		

OVERVIEW STUDY PLAN

Consecutive endometrial cancer patients of all preoperative risk groups with the primary tumor confined to the uterus will be screened for inclusion.

↓

Inclusion of eligible consenting women. Distribution of a validated questionnaire on lymphedema.

↓

Four point submucosal cervical injection of 4 x 0.245mL, 2,5 mg/mL ICG (Indocyanine Green) solution at onset of surgery.

↓

Pelvic SLN identification by Near Infrared Fluorescent technology with ipsilateral reinjection of 0.25 mL ICG solution in case of non-mapping of either of the right or left upper paracervical (UPP)-pathways.
Removal of identified SLNs according to definitions including a separate removal of the ParaUterine Lymphovascular Tissue (PULT).
Removal of SLNs along the LPP in high risk histologies (Non-endometrioid cancers).
SLNs will not routinely be sent for frozen section.

↓

Registration of SLN- associated intraoperative adverse events. Registration of time for the SLN procedure and related complications.

Execution of an infrarenal paraaortic lymphadenectomy in women with FIGO grade 3 endometrioid histology or Non-endometrioid histology and >= 50% myometrial invasion deemed at preoperative ultrasonography or MRI.

Completion of a Querleu Morrow B1-B2 to C2 robotic hysterectomy and BSO. Infracolic omentectomy in non-endometrioid histology.

SLN's and PULT for ultrasectioning and immunohistochemistry. Paraaortic nodes for standard pathological bisectioning and staining with hematoxylin/Eosine.

Registration of postoperative adverse events until 30 days after surgery using the Clavien Dindo classification and SAE using defined criteria and log.

Continuous data management. Analysis of technical success (mapping) rates per pathway. Analysis of positions of SLNs and metastatic SLNs per major pathways and per subcompartment. Analysis of subtimes and complications relate the SLN procedure.

↓

Analysis of size of SLN metastases (macrometastases, micrometastases and isolated tumor cells as of below) subdivided by pre and postoperative risk group criteria. Analysis of the relation of biomarkers related nodal status

↓

Clinical follow up with interval according to national guidelines. A vaginal ultrasonography shall be performed at each visit for detection of pelvic lymphoceles. Clinical investigation of leg and/or trunchal lymphedemas.

↓

Mail distribution of a validated questionnaire on lymphedema for comparison with the same preoperative questionnaire for evaluation of new onset lymphedema .

Table of contents

1. OBJECTIVES
2. BACKGROUND
3. PATIENT AND SURGEON ELIGIBILITY
4. STUDY MODALITIES
5. EVALUATION CRITERIA
6. DURATION OF STUDY
7. STATISTICAL CONSIDERATIONS
8. STUDY MONITORING AND REPORTING PROCEDURES
9. BIBLIOGRAPHY

1. OBJECTIVES

To estimate mapping rates and exact anatomic positions of SLNs and metastatic SLNs with information on size of SLN metastases (macrometastases, micrometastases and isolated tumor cells) in patients with endometrial cancer of all preoperative risk groups with the primary tumor confined to the uterus planned for robot assisted surgery. Indocyanine green (ICG) will be used as tracer, the Da Vinci Near Infrared Imager (Fire Fly®) will be used for visualization of ICG. A previously developed and published anatomically based surgical algorithm and definitions of pelvic SLNs will be adhered to.

- To estimate the proportion of endometrial cancer patients feasible for the SLN ICG concept.
- To estimate complications associated with ICG as tracer.
- To estimate intraoperative complications associated with the detection and removal of SLN as such subdivided by experience of surgeon.
- To estimate time used for SLN detection subdivided by experience of surgeon to establish a learning curve.
- To estimate the incidence of lymphatic complications (lower limb and truncal lymphedema and lymphoceles) by the use of a validated questionnaire and a structured follow up including vaginal ultrasonography in all women evaluated a minimum 12 months postoperatively.

From November 2020 to estimate the proportion of nodal metastases in non-mapped nodes at typical positions along the UPP regardless of ICG-mapping elsewhere along the ipsilateral UPP. We defined typical positions as the “proximal obturator fossa” (lateral of the obliterated umbilical area, dorsomedial of the ventral rim of the external iliac vein, ventral of the obturator nerve and distal of the first part of the internal iliac vein extending one third of the obturator fossa). Furthermore, the “interiliac position” lateral of the obliterated umbilical

artery, ventromedial of the external iliac vein, within the bifurcation of the external and internal iliac arteries. Hence, external iliac ICG defined SLNs lateral of the external iliac artery wherever longitudinally positioned were considered atypically positioned.

2. BACKGROUND

In endometrial cancer, nodal involvement is a strong prognostic factor for recurrence and survival and also determines adjuvant treatment. Historically, a pelvic lymphadenecomy is generally recommended for high risk cancers whereas low risk cancers usually are not subject for any lymphadenectomy. A pelvic lymphadenectomy is associated with an approximate 20% risk for lymphatic complications such as chronic lymphedema, lymphocysts and in rare cases lymphatic ascites.

The concept of excluding pelvic nodal involvement by identifying nodal metastases by a restricted nodal removal by detection of sentinel lymph nodes therefore is appealing. During later years many studies have been published, most of them retrospective, using a variety of tracers and with little or no information on used surgical algorithm, used definitions of SLNs and without reference to lymphatic anatomy. Consequently, technical success rate/mapping rate varies. A high mapping rate is crucial to avoid the need for a full hemipelvic or pathway LND in case of failure, so far recommended. In pilot studies from our institution using ICG as tracer reinjection results in bilateral mapping rates, defined by detection of at least one SLN per hemipelvis of 95%. ICG has also been proven superior to other tracers in a separate study. By the use of ICG a fluorescent tracer, we have also previously demonstrated two bilateral separate pelvic pathways;

-The Upper Paracervical Pathway (UPP) running along the PULT to external iliac and/ or obturator nodes and further lateral to the common iliac artery to paraaortic node.

-The Lower Paracervical Pathway (LPP) running via the sacrouterine ligaments to nodes medial of the internal iliac artery and/ or presacral nodes then further medial to the common iliac artery to paraaortic nodes.

Hence there are two bilateral pelvic pathways draining further to the paraaortic region both of which basically should be evaluated for SLNs independently.

This study aims to evaluate the pelvic sentinel node concept based on a defined surgical algorithm, using ICG as tracer, allowing reinjection, and with a clear definition of SLNs based on described uterine lymphatic pathways. Surgical competence and experience is necessary to achieve a high technical success rate and a low false negative rate for SLN. The study setting including only high volume surgeons at one high volume center enables an evaluation of the potential of the pelvic SLN concept.

3. PATIENT AND SURGEON ELIGIBILITY

Inclusion Criteria

- Age 18 years and older at the time of informed consent.

- A histologically proven EIN or endometrial carcinoma of any histologic subtype with the primary tumor confined to the uterus.

-Absence of any exclusion criteria

-Written consent to participate in the study

Exclusion Criteria

-Non-consenting patients

-Ongoing pregnancy

-Inability to understand written and/or oral study information

-WHO performance status III or more

-Previous lower limb lymphedema

-Surgical contraindication to a laparoscopic/ robotic approach or lymphadenectomy at surgeons discretion.

-Anesthesiologic contraindication to a laparoscopic approach at the anesthetist's discretion

- Locally advanced disease or intraabdominal/distant metastases at preoperative CT, MRI or ultrasonography.

- Radiologically suspect pelvic nodal metastatic disease according to the RECIST criteria (>= 1 node with >=16 mm short axis diameter).

-Allergy to Iodine.

-Patients with an acute or chronic liver disease.

-Patients with a significant bleeding disorder or mandatory antithrombotic treatment

Surgeon eligibility

All included surgeons at the investigating center were instructed and approved by the principal investigating surgeon (JP) by participating in at least five of the first SLN-procedures per surgeon. All included surgeons must have a previous experience of at least 100 oncologic robot assisted procedures.

4. STUDY MODALITIES

Surgical Procedures

Patients with any subtype of endometrial cancer with the primary tumor deemed confined to the uterus planned for primary surgery with a Querleu-Morrow B1 – C1 robotic hysterectomy with the Fire-Fly modality for detection of ICG-uptake will be approached for the study.

A submucosal cervical injection of 0,25 mL 2.5m/mL ICG solution at 2-4-8-and 10 O'clock respectively (totally 1 mL) will be performed at immediate onset of surgery.

The pelvic sidewalls and the presacral area will be inspected, transperitoneally or if needed, retroperitoneally, for evaluation of ICG uptake per pathway (UPP only in endometrioid cancers, and LPP in high risk histology cancers bilaterally). Ipsilateral cervical reinjection of 0.25 mL of the ICG solution at 3 or 9 O'clock in case of non-display of the UPP

Detection and removal of SLN will follow a strict protocol with a proximal- presacral dissection before the distal external iliac-obturator nodal compartment dissection and with careful opening of the presacral, paravesical and pararectal planes to avoid leaking of tracer. SLNs will be defined as described below.

The described procedures in this protocol will be performed with robot-assisted laparoscopy, or via the open surgery wound utilizing the Da Vinci robot and FireFly camera and with removal of SLN and non-SLN's before the radical hysterectomy.

Histopathologic assessment.

The department of pathology was coordinated regarding principles for management of frozen section and later ultrasectioning and immunohistochemistry of SLNs and PULT and management on non-sentinel lymph nodes.

Drug Information:

Description: ICG is a sterile, lyophilized green powder containing 25 mg of Indocyanine green with no more than 5% sodium iodide. The ICG is prepared by thorough mixing of 10mL of sterile water with the lyophilized ICG in its vial creating a 2.5mg/mL concentration. The lot number, expiration date and dose injected (mg) will be recorded. The ICG solution is prepared immediately before surgery and intended for single patient use. The ICG solution is stored at room temperature covered to prevent illumination before use. The solution is active for 6 hours and should be discarded after that period of time. Manufacturer: Pulsion medical system, Feldkirchen Germany Availability: ICG will be provided by the manufacturer. Please refer to the current ICG package insert for complete prescribing information

Adverse Effects if ICG: All known adverse effects are allergic in nature and occur in <1% of patients. Anaphylactic or urticarial reactions have been reported in patients with or without a history of allergy to Iodine. If such reaction occur standard treatment with appropriate agents (e.g. adrenalin, antihistamines and corticosteroids) will be initiated.

Contraindications: known hypersensitivity to iodine containing compounds. Known liver failure. Radioactive iodine uptake studies should not be performed for at least 1 week following the use of ICG.

Injection of Indocyanine Green (ICG)

Two separate sterile 1mL syringes is prepared with 1 mL ICG solution (2.5mg ICG) in each syringe.

A 0.6x38 mm 23G needle is attached to each syringe for the injection. A separate back table is used for the syringes.

The ICG injection will be performed immediately before placement of surgical ports and docking the robot or before opening the abdomen, hence allowing approximately five minutes for distribution of ICG.

ICG volume is injected submucosally 2-4-8-and 10 O'clock respectively to a total dose of 2.5mg ICG and a total volume of 1 mL.

After injection of dye, a compression free fornix presenter without an intracervical device is placed. A second ipsilateral injection of 0,5 mL ICG is performed in case of non-display either of the upper (UPP) or lower (LPP) paracervical pathways after a 10 minutes observation time after ICG injection. The injection is done at 3 and 9 O'clock respectively. Display of the separate lymphatic pathways (UPP, LPP will be recorded after the first, and if performed, after the second injection).

Sentinel Node Identification

The bilateral technical success rate of SLN identification is important to determine if this technique can be transferred to clinical practice. A lower bilateral detection rate will lead to the need of full lymphadenectomy in a higher proportion of patients. We intend to separately study the extent of enhancement of bilateral detection rates as a result of reinjection.

Definitions of SLNs

A clear anatomically based definition of what is a SLN and a strict surgical algorithm as

described in this study is important.

The SLNs are defined as the juxtauterine ICG positive nodes with an afferent ICG positive lymphatics in each of the UPP and LPP respectively bilaterally with the potential of parallel lymphatics in the UPP to the external and obturator areas. These SLNs are defined as **SLN type 1.**

In case of a ICG positive lymph vessel where no nodes are ICG positive in that pathway, the node where the ICG positive lymphatic channel ends is defined as **SLN type 2**.

Importantly, as the **PULT** represents the lymphatic pathway between the uterus an lateral pelvic nodes, removal of the PULT is considered a part of assessment of the lymphatic system.

SLNs type 1 and 2 and PULT will be used for evaluation of the SLN-ICG algorithm

Nodes macroscopically suspect of metastatic disease will be defined as **SLN Macro** regardless of ICG uptake but with information on ICG-uptake.

In case of no ICG- display along the LPP a full presacral lymphadenectomy is performed and the nodes defined presacral **SLN anatomy-LPP**

Along the UPP, in case of lack of ICG positive nodes at either frequent position (interiliac and proximal obturator) visible nodes at those positions were removed and also defined as "**SLN anatomy"** with reference to anatomic position. This amendment was added in November 2020.

All types of SLNs will be used for evaluation of the overall SLN algorithm.

Importantly, to secure accuracy, the positions and types of SLNs will be marked on an anatomical chart, recorded on a list with anatomical locations and placed in pre-labeled jars with corresponding names and numbers. This list is used by the department of pathology for reporting the results to minimize the risk of errors in location of nodes and which nodes are SLN's, the types of SLN as of above and non-SLN's. A copy of the list is kept in the patients' study file. Nodes defined as SLNs will have red labels on the jars,

other nodes will have black labels.

**Anatomical boundaries of lymph node compartments in the female pelvis**				
**Lymph node compartment**	**Proximal limit**	**Lateral limit**	**Distal limit**	**Medial limit**
External iliac area	Bifurcation of external and internal iliac artery	Genitofemoral nerve	Cloquets lymph node	External iliac vein
Obturator fossa	Internal iliac vein	Ileopsoas muscle	Os pubis, obturator nerve	Obliterated umbilical artery
Common iliac	Aortic bifurcation	Genitofemoral nerve Psoas muscle	Bifurcation of external and internal iliac artery	Common iliac artery
Presacral	Aortic bifurcation	Common iliac artery	Lower promontory	Hypogastric nerve (as distinction between right and left)
Lower paraaortic	Inferior mesenteric artery	Ureter	Aortic bifurcation	
Higher paraaortic	Left renal vein	Ureter	Inferior mesenteric artery	

Anatomic description of lymphatic pathways draining the uterus

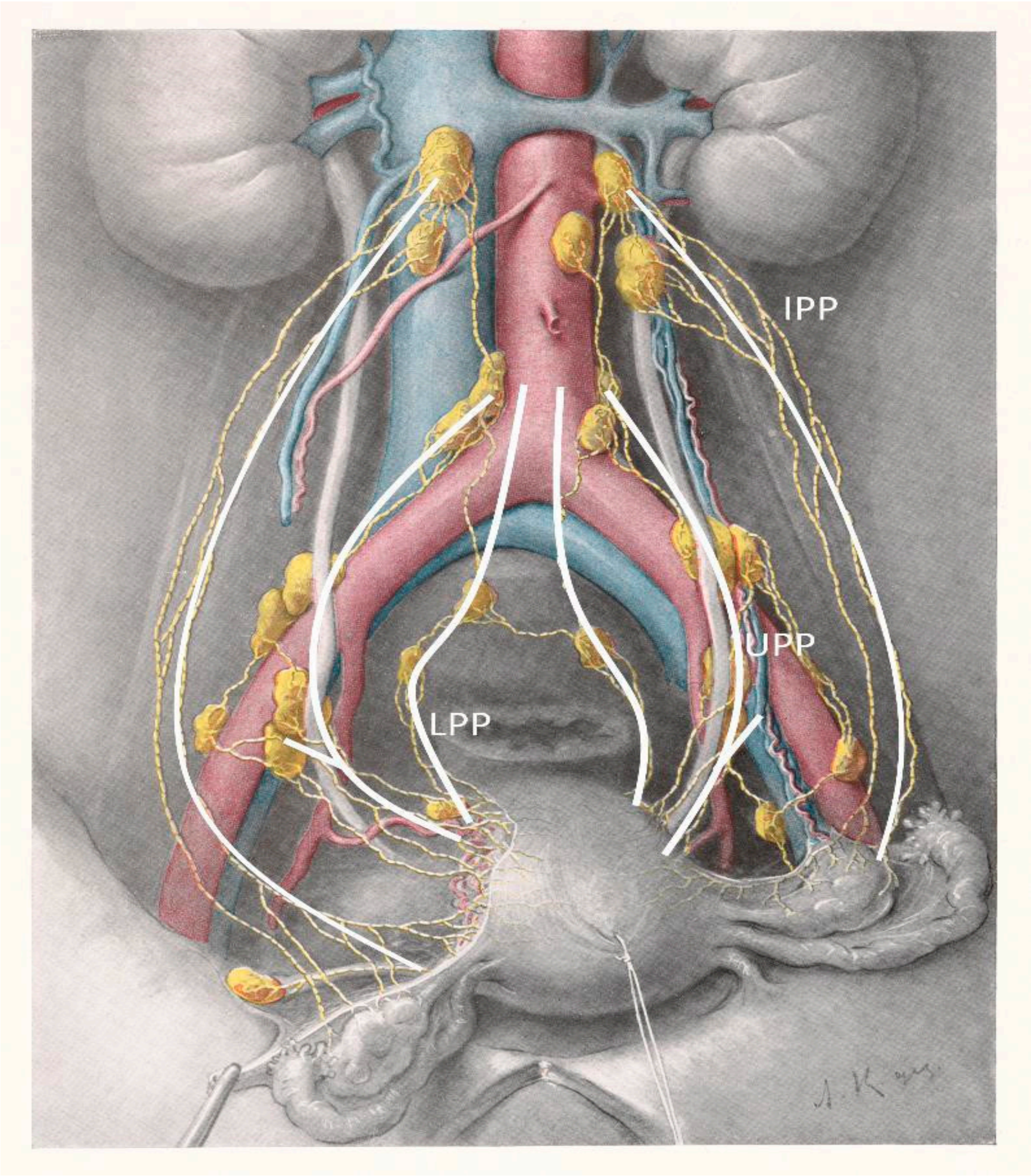

The upper paracervical pathway (UPP) follows the uterine artery (via the Parauterine Lymphovascular Tissue; PULT) to the pelvic side wall draining primarily to the external iliac and obturator nodal compartments, then running lateral to the common iliac artery further to the paraaortic area.

The lower paracervical pathway (LPP) follows the ventral rim of the sacrouterine ligament, primarily to internal iliac and presacral nodes, then running medial of the common iliac artery further to the paraaortic area.

For the pelvic SLN concept, ideally one SLN should be identified per left and right LPP and at least one SLN per left and right UPP (parallel lymphatics to the obturator and external areas are frequent) in addition to any nodes in the left and right PULT.

The infundibulopelvic ligament pathway (IPP) runs via the IP-ligament further to the supramesenteric, infrarenal paraaortic area.

Logically, assuming that lymph runs from the uterus and then cranially, supramesenteric true paraaortic SLN's can be defined only when inframesenteric lymph are not ICG positive: Theoretically this may occur in situation when no pelvic nodes are dyed or when ICG have dyed pelvic nodes but not have been distributed further to the inframesenteric node, i.e ICG goes directly to the supramesenteric nodes via the IP- ligament.
Alternatively, when inframesenteric paraaortic nodes are ICG positive via the UPP and /or the LPP but no pelvic nodes in these pathways are dyed, a situation unlikely to occur as juxtauterine SLN along those patways almost invariably are identified.

Given these prerequisites, paraaortic SLNs rarely can be defined, therefore not included in the present study.

Anatomy chart for positioning of pelvic sentinel lymph nodes and their classification and protocol for ICG mapping.

ICG-uptake 1:st injection:

	UPP	LPP	IP-ligament
Right			
Left			

Reinjection cervix: ☐ yes ☐ no

	UPP	LPP	IP-ligament
Right			
Left			

Typical position
Externa right ☐ yes ☐ no
Oburator right ☐ yes ☐ no

Typical position
Externa left ☐ yes ☐ no
SLN obt vä ☐ yes☐ no

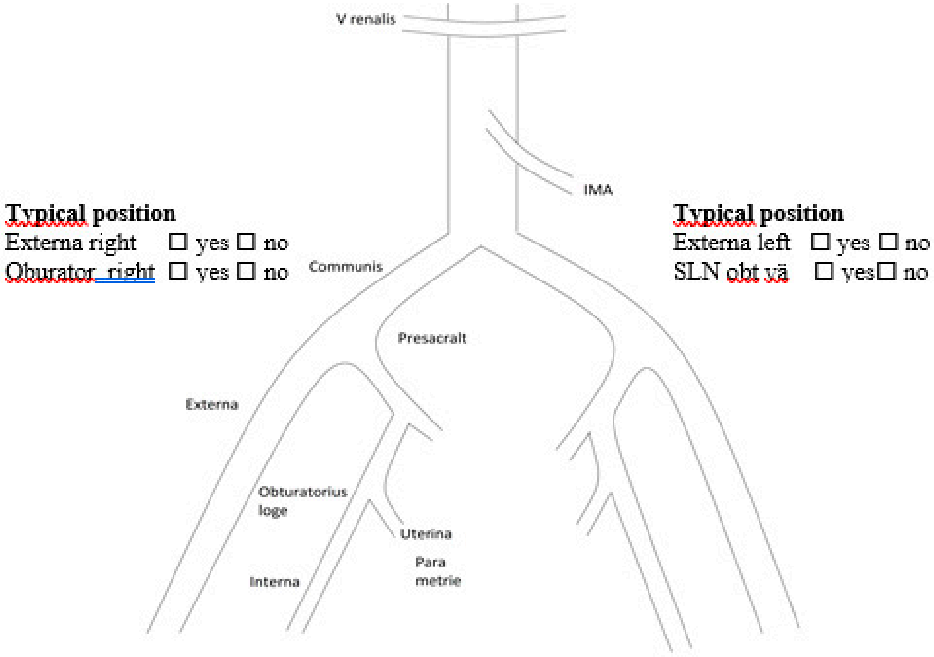

Mark the position of each SLN per definitions in the sketch with the assigned figure and number on the specimen list.

Figures:

○ = ICG positive Sentinel node (**SLN1**) with an afferent lymphatic channel

☐ = ICG negative juxtauterine node with an afferent lymphatic channel (**SLN 2**)

Δ = SLN anatomy

X = Tumor suspect node (**SLNmakro**) (note in specimen list if ICG pos or not)

List of nodal specimens.

OB&G Jar Nr	Nodal position	Pathology Jar Nr	Number of nodes by histology	Of which metastatic
1	Non SLNs external right ICG neg			
2	Non SLNs external right ICG pos			
3	Non SLNs obturator right ICG neg			
4	Non SLNs obturator right ICG pos			
5	Non SLN common right ICG neg			
6	Non SLN common right ICG pos			
7	Non SLNs external left ICG neg			
8	Non SLNs external left ICG pos			
9	Non SLNs obturator left ICG neg			
10	Non SLNs obturator left ICG pos			
11	Non SLN common left ICG neg			
12	Non SLN common right ICG pos			
13	Non SLNs presacral left ICG neg			
14	Non SLNs presacral left ICG pos			
15	Non SLNs presacral right ICG neg			
16	Non SLNs presacral right ICG pos			
17	Inframesenteric nodes ICG neg			
18	Inframesenteric nodes ICG pos			
19	Infrarenal nodes ICG neg			
20	Infrarenal nodes ICG pos			
21	PULT right			
22	PULT left			
23	SLN type 1 presacral right			
24	SLN type 1 presacral left			
25	SLN type 1 external right			
26	SLN type 1 obturator right			
27	SLN type 1 external left			
28	SLN type 1 obturator left			
29	SLN type 1*			
30	SLN type 1*			
31	SLN type 2*			
32	SLN type 2*			
33	SLN macro ICG pos*			
34	SLN macro ICG neg*			
35	SLN anatomy*			
36	SLN anatomy*			
37	SLN anatomy*			
38	SLN anatomy*			
39	SLN anatomy*			
40	SLN anatomy*			

*Numbers 29-40 will be used for describing locations outside defined typical positions and for SLNs t ypes 2, SLN macro and SLN anatomy as appropriate. The locations will be written by hand on list and on labels for jars.

Histopathologic evaluation SLNs/PULT and non-SLNS

At final pathological examination, all macroscopically identified SLNs /lymphoid tissue will be embedded and bisected if the minimum thickness exceeds 3 mm. Ultrastaging using hematoxylin/ Eosin staining will be performed in five sections at three different levels , separated by 200 μm. First and second level immunohistochemistry (pan-cytokeratin, MNF 116) will be performed if the maximum diameter of the SLN tissue exceeds one mm. If no macroscopically lymphoid tissue is identified in SLN or parametrial tissue the most suspicious find will be embedded and microscopically investigated. ITCs can be detected by Hematoxylin/Eosin or by Immunohistochemistry alone.

Non- SLN nodes less than 3 mm in thickness will be embedded whole, and from nodes thicker than 3 mm at least half the node will be embedded. The slides will be evaluated after hematoxylin/Eosin staining.

Classification of tumor size in SLN's

Sentinel nodes will be classified according to a modification of the AJCC staging for axillary nodes from breast cancer as follows:

Macrometastases = tumor greater than 2.0 mm in diameter.

Micrometastases = tumor cell aggregates between 0.2 and 2.0 mm in diameter.

Isolated tumor cells =individual tumor cells or aggregates that are less than 0.2mm in diameter, usually detected by immunohistochemistry.

Tumor absent – no tumor cells identified in H&E (or immunohistochemically, if applicable) stained sections.

Non-sentinel lymph nodes will be reported as positive or negative for metastases based upon routine sectioning and examination of a single H&E stained section.

Surgical protocol

Consolidation study Sentinel node in endometrial cancer CRF number (by study coordinator)

Surgical protocol

Date
Surgeon………………………Assisting surgeon……………………..
OR nurse……………………..Circulating nurse……………………….

Included as/ planned surgery

☐ Low risk endometrial cancer. SLN only

☐ High risk endometrial cancer, SLN only

☐ High risk histologyendometrial cancer, SLN+ paraaortic LND due to preop MI >50%

Reason for an intraoperative deviation from a planned surgery

____

**Previous surgery**	1	☐ No
	2	☐ App
	3	☐ Pfannenstiel
	4	☐ Midline upper
	5	☐ Midline lower
	6	☐ Laparoscopy/ robot
	7	☐ other, specify
**Vikt (kg)**		
**Längd (m)**		

**Surgery**		**(specify all)**
	1	☐ Radical hysterectomy+adnex , QM type
	2	☐ Enkel hysterektomi+ adnex
	3	☐ Sentinel node UPP +LPP
	4	☐ Sentinel node UPP only
	5	☐ Full pelvic LND
	6	☐ Paraaortic LND to LRV
	7	☐ Paraaortic LND to IMA
	8	☐ Omentectomy
		☐ Other specify

**Pat in OR**	time.
**First Dr's procedure**	time
**Time for SLN Onset dissection incl reinj**	Minutes:
**Last stich**	time
**Pat out OR**	time

Uterus weight (g)

Insufflation technique
☐ Palmer point direct entry
☐ Hasson periumbilical
☐ Veres needle

Adhesiolysis before onset of surgery
☐no
☐Yes
Total minutes ___ for adhesiolysis (before and after docking)

Nr robot instuments
☐2
☐3
☐4
☐5
☐6

Assistant trocar

☐1	☐ 12mm ☐ disp ☐ non-disp
☐2	☐ 15 mm ☐ disp☐ non-disp
☐3	☐ 18 mm ☐ disp ☐ non-disp
☐4	☐ 5 mm ☐ disp ☐ non-disp

Additonal instruments
☐ Endobag,nr____________
☐ Tachyseal,/ floseal, nr___________________
☐ Other specify_________________________

**Bleeding**	ml____________________
**Transfusion**	☐ No<br>☐ Yes nr units:_____________________

′

**Conversion**	☐no<br>☐Yes to laparotomy
**Cause of conversion**	☐ Robot, technical problem,; specify:____________________<br>☐ Surgery, specify:______________________________<br>☐ Anesthesiologically cause; specify:____________________<br>☐ Oncological cause; specify:____________________
**Complications during insufflation or adhesiolysis**	☐ None<br>☐ **Yes. Specify:**________________________________
**Complications during SLN removal**	☐ No<br>☐ **Yes. Specify**:______________________________
**Complications during remaining pelvic LND**	☐ No ☐ Not performed<br>☐ **Yes. Specify**:______________________________
**Complications during remaining paraaortic LND**	☐ No ☐ Not performed<br>☐ **Yes. Specify**:______________________________
**Complications during hysterectomy**	☐ No ☐ Not performed<br>☐ **Yes. Specify**:______________________________

**Technique for removal of uterus**	☐ vaginally without bag ☐ vaginally with Endobag<br>☐ Though abdominal wall in bag, specify why and where.<br>____
**Technique for removal of nodes**	☐ Trough assistant port in bag/container<br>☐ vaginally in bag
**Nodal tissue divided in abdomen to an enable removal through port?**	☐ No ☐ yes specify
**Closure of fascia**	☐ Assistant port<br>☐ Optics port (SI robot)<br>☐ Other ports, specify;________________________________

STUDY CRITERIA

Schedule of events table/ checklist

Procedures	Screening	Enrollment/ Baseline	Surgery-ICG SN/postoperative parameters	Final histology Follow up
**Medical History routine**	X			
**Gynecologic History routine**	X			
**Physical / lab exam routine**	X			
**CT abdomen/chest evaluation**	X			
**Vaginal US evaluation**	X			
**Informed consent oral**		X		
**Informed consent written**		X		
**Enrollment**		X		
**Preop CRF incl lymphprotocol**		X		
**Injection data for ICG**			X	
**SLN detection and removal incl protocols**			X	
**Recording of intraoperative SLN related adverse events and times**			X	
**Histologic evaluation of sentinel nodes and non-sentinel nodes**				X
**Evaluation of size of metastases. their relation to pre-postop risk groups**				X

Procedures	Screening	Enrollment/ Baseline	Surgery-ICG SN/postoperati ve parameters	Final histology Follow up
**Postop CRF/including 30 day AE/SAE record**				**X**
**Continuous clinical follow up Vaginal US Recurrence Clinical Lymphedema**				**X**
**Second Lymph protocol distributed 1-2years after surgery**				**X**

5. EVALUATION CRITERIA

Mapping rate with ICG will be recorded per patient, per hemipelvis and per pathway, UPP and LPP respectively before and after reinjection.

Mapping of types of SLNs per sub-compartment with information of anatomic localization subdivided as typical or not typical per protocol.

Registration of intraoperative adverse events by subparts, including the SLN part. Registration on the separate time for SLN detection and removal per surgeon. Registration of time for the procedure.

Intraoperative adverse events related to study drug (ICG) will be evaluated and reported to safety monitoring committee.

Postoperative complications until 30 days after surgery will be reported and if grade III or more evaluated in terms of a potential association with study drug or SLN procedure.

Evaluation of size of SLN metastases (macrometastase, micrometastases and isolated tumor cells) per pre- and postoperative uterine stage and histologic subtypes.

Evaluation of the association between metastatic SLNs and selective biomarkers.

Evaluation of lymphatic complications (pelvic lymphoceles by Vaginal US at each follow up), clinical signs of limb or trunchal lymphedema at each follow up. Use or pre- and postop Lmpn QOL evaluated questionnaire.

Evaluation of 3 and 5 year recurrence by histology, uterine stage and nodal status.

6. DURATION OF STUDY

The patient may withdraw from the protocol at any time prior to surgery or at any time until retrieval of postoperative data.

The study is initiated at Skåne University hospital, Lund, and intended to be a single institution study for congruency and bias-minimizing reasons, in particular as control of individual surgical skill is difficult.

As no distinct sample size analysis can be performed, this study is planned to go on until at least 1500 women are included as some subparts may need a larger number of included women than other subparts. The stud can be considered hypothesis generating in several aspects. The study also acts as a continuous interal quality assessment. The study complies with the national treatment protocol for endometrial cancer why an extension is considered ethically aceeptable.
A continuous evaluation of the duration of the study will be performed based on the respective parameters.

7. STATISTICAL CONSIDERATIONS

This is an observational non-randomized cohort, partly hypothesis generating, study where no sample size analysis, distinct interval analysis time or elaborated statistical analyses is needed. Moreover, the study covers several aspects with different need for case volume.

8. STUDY MONITORING AND REPORTING PROCEDURES

ADVERSE EVENT REPORTING

Definitions

An adverse event (AE) is any new medical problem or exacerbation of an existing problem experienced by a subject enrolled in the study, whether or not it is considered drug-related by the investigator.

This study will utilize the Adverse Events Logs (Tables S2–S4). Any SAE will be reported to the study coordinator michele.bollino@med.lu.se or jan.persson@med.lu.se utilizing the SAE Events Log outlined below.

Adverse events related to the study drug (ICG).
All adverse events occurring from the first dose of study drug until hospital discharge (whether or not attributed to the study drug) will be reported on the Adverse Event Log. In addition, any adverse event reported by the subject to the investigator after discharge and determined to be reasonably associated with the study drug should also be captured and followed until resolution.

Adverse events related to the sentinel node procedure as such (excluding AE related the study drug, ICG)

All intraoperative events related to the SLN procedure will be reported on the adverse events log.

Adverse events related the surgical procedure (excluding the SLN part) including AE until 30 postoperative days.

All adverse events will be reported on the adverse events logs.

Serious adverse event (SAE):

An adverse event that results in one or more of the following:

-Any death occurring prior to the postoperative outpatient evaluation 30 days postoperatively.

-Any life-threatening event until and including 30 postoperative days.

-Any medical event requiring inpatient hospitalization or prolongation of existing hospitalization beyond five postoperative days

NOTE: Hospitalizations that are not considered SAE are:

-Hospitalization planned prior to first administration of study drug

-Hospitalization for elective treatment of a pre-existing condition unrelated to the study medication

- Hospitalization due to social / practical reasons such as an untimely coordination with local community home care services.

Attribution: Attribution is the determination of whether an adverse event is related to a medical treatment or procedure. The categories of attribution are:

Definite: The adverse event is clearly related to the study drug Probable: The adverse event is likely related to the study drug. Possible: The adverse event may be related to the study

drug. Unlikely: The adverse event is doubtfully related to the study drug. Unrelated: The adverse event is clearly NOT related to the study drug.

Unexpected Adverse Event: An unexpected adverse event is an event not mentioned in the package insert/ manufacturer's instructions or the specificity or severity of which is not consistent with the package insert/ manufacturer's instructions.

The grading described beneath and the attribution described above will be used for cathegorization of unexpected adverse events.

Copies of all serious adverse event reports will be kept on file the department of Obstetrics and Gynecology, Lund University Hospital.

The study coordinator will also report all individual SAE's related to study drug (ICG) , the sentinel node procedure as such, are life threatening or resulting in death (as defined) to the Safety Monitoring Committee (SMC) for clinical studies at Skåne University Hospital for an independent evaluation.

At any time during the conduct of the trial, if it is the opinion of the investigator that the risks (or benefits) to the patient warrant early closure of the study, this recommendation should be made in writing to the SMC. Alternatively, the SMC may initiate suspension or early closure of the study based on its review of the investigator reports.

Study Monitoring / study accrual oversight

Apart from monitoring described below, the number of node positive patients and potential false negative SLN's will be monitored continuously by e-mail to the study coordinator by the use of the study number assigned to each patient.

In case of identified inconsistencies or missing data, additional source documents (identified only by unique patient number) will be requested from the site to resolve ongoing inconsistencies.

Data Management

Preoperative, intraoperative and postoperative data from each surgery will be recorded on the standardized study sheets. Each patient will be allocated a unique study number which will serve as the prefix to the case number.

The investigating center will hold a record with the full identification of patients whereas data otherwise should only identify the patient by the study number (see above).
Upon interim analysis the full data will be monitored by the study coordinator, the principal investigator and study statistician.

Early Study Closure

Death will be reported according to section 10.1 above and per local IRB reporting guidelines. The SMC will review all reported deaths monthly. Early closure of the study will be based on judgement of the SMC.
The study will be stopped for futility reasons as described under the statistics section.

Protocol Deviations

Major protocol deviations shall be reported by to the study coordinator michele.bollino@med.lu.se, or Jan.persson@med.lu.se and filed using the designated study number. Major protocol deviations include, but are not limited to, violations to inclusion/ exclusion criteria, erroneous preparation of ICG or surgery by a non-accredited surgeon

SAE events log

Per definitions of SAE and attributions as outlined in protocol

**Patients' CRF number**<br>**Date for SAE**		
**Type of SAE**	**Yes /no**	**Attribution**
**Death**		
**Life Threatening**		
**Drug /ICG related**		
**Intraoperative related the**<br>**SLN procedure as such**		
**Intraoperative related the**<br>**full LND/ hysterectomy**		
**Postoperative**		
**Unexpected AE**		

Description / outcome of the SAE

Intraoperative Adverse events will be graded according to the following scale:

Grade	Description
**Grade 1**	Mild; asymptomatic; not interfering with function.
**Grade 2**	Moderate; symptomatic; interfering with function but not ADL; medical intervention indicated.
**Grade 3**	Severe; symptomatic; interfering with ADL; operative intervention indicated; IV intervention indicated
**Grade 4**	Life-threatening; major urgent intervention indicated; disabling.
**Grade 5**	Death

Table S2. Adverse Events related injection of ICG (examples).

CRF	Description AE /SAE	Grade
NN	Mild allergic reaction<br>(AE)	2

Table S3. Adverse Events related SLN dissection and removal (examples).

CRF	Description AE/SAE	Grade
NN	Obturator nerve damage<br>(AE)	3

Postoperative adverse events will be graded according to the Clavien Dindo classification

Grade I	Any deviation from the normal postoperative course without the need for pharmacological treatment or surgical, endoscopic and radiological interventions Allowed therapeutic regimens are: drugs as antiemetics, antipyretics, analgetics, diuretics and electrolytes and physiotherapy. This grade also includes wound infections opened at the bedside.
Grade II	Requiring pharmacological treatment with drugs other than such allowed for grade I complications.<br>Blood transfusions and total parenteral nutrition are also included.
Grade III	Requiring surgical, endoscopic or radiological intervention
- IIIa	Intervention not under general anesthesia
- IIIb	Intervention under general anesthesia
Grade IV	Life-threatening complication (including CNS complications)* requiring IC/ICU- management
- IVa	single organ dysfunction (including dialysis)
- IVb	Multi organ dysfunction
Grade V	Death of a patient

Table S4. Postoperative (<=30 days) adverse Events. Clavien Dindo Grade 1excluded.

**CRF**	**Description AE/SAE**	**Clavien Dindo Grade**
NN	Readmisson, vaginal cuff infection, drainage.	3

9. BIBLIOGRAPHY

1. Persson J, Geppert B, Lönnerfors C, Bollino M, Måsbäck A. Description of reproducible anatomically based surgical algorithm for detection of pelvic sentinel nodes endometrial cancer. Gynecol Oncol 2017 Gynecol Oncol. 2017 Oct;147(1):120-125. doi: 10.1016/j.ygyno.2017.07.131. Epub 2017 Jul 24

2. Geppert B, Lönnerfors C, Bollino M, Arechvo A, Persson J. A study on lymphatic anatomy for standardization of pelvic sentinel lymph node detection in endometrial cancer, Gynecol Oncol 2017. May; 145(2):256-261. Doi 10.1016/ j. yfyno. 2017.02.018. Epub 2017 feb 10.

3. Frumovitz M, Plante M, Lee PS, Sandadi S, Lilja JF, Escobar PF, Gien LT, Urbauer DL, Abu-Rustum NR. Near-infrared fluorescence for detection of sentinel lymph nodes in women with cervical and uterine cancers (FILM): a randomised, phase 3, multicentre, non-inferiority trial.Lancet Oncol. 2018 Oct;19(10):1394-1403. doi: 10.1016/S1470-2045(18)30448-0. Epub 2018 Aug 22.PMID: 301434

4. Jan Persson , Sahar Salehi , Michele Bollino , Celine Lönnerfors , Henrik Falconer , Barbara Geppert . Pelvic Sentinel lymph node detection in High-Risk Endometrial Cancer (SHREC-trial)-the final step towards a paradigm shift in surgical staging Eur J Cancer . 2019 Jul;116:77-85. doi: 10.1016/j.ejca.2019.04.025. Epub 2019 Jun 7.

5. Geppert B, Lönnerfors C, Bollino M, Persson J. Sentinel lymph node biopsy in endometrial cancer. Feasibility, safety and lymphatic complications. Gynecol Oncol. 2018 Mar;148(3): 491-498. doi:10.1016/j.ygyno.2017.12.017. Epub 2017 Dec 20.PMID: 29273307

6. Lührs O, Ekdahl L, Lönnerfors C, Geppert B, Persson J. Combining Indocyanine Green and Tc99-nanocolloid does not increase the detection rate of sentinel lymph nodes in early stage cervical cancer compared to Indocyanine Green alone. Gynecol Oncol. 2020 Feb;156(2):335-340. doi: 10.1016/j.ygyno.2019.11.026. Epub 2019 Nov 26. PMID: 31780237
